# Kinematic ME-MAFA for Pseudolite Carrier-Phase Ambiguity Resolution in Precise Single-Point Positioning

**DOI:** 10.3390/s20216197

**Published:** 2020-10-30

**Authors:** Kai Liu, Xiye Guo, Jun Yang, Xiaoyu Li, Changshui Liu, Yuqiu Tang, Zhijun Meng, Enqi Yan

**Affiliations:** College of Intelligence Science and Technology, National University of Defense Technology, Changsha 410000, China; liukai14a@nudt.edu.cn (K.L.); yangjun@nudt.edu.cn (J.Y.); lixiaoyu14@nudt.edu.cn (X.L.); lcscsu@126.com (C.L.); tangyuqiu168@126.com (Y.T.); mengzhijun@nudt.edu.cn (Z.M.); yanenqi@nudt.edu.cn (E.Y.)

**Keywords:** pseudolite system, single-point positioning, carrier-phase ambiguity resolution, modified ambiguity function approach

## Abstract

Precise single-point positioning using carrier-phase measurements can be provided by the synchronized pseudolite system. The primary task of carrier phase positioning is ambiguity resolution (AR) with rapidity and reliability. As the pseudolite system is usually operated in the dense multipath environment, cycle slips may lead the conventional least-squares ambiguity decorrelation adjustment (LAMBDA) method to incorrect AR. A new AR method based on the idea of the modified ambiguity function approach (MAFA), which is insensitive to the cycle slips, is studied in this paper. To improve the model strength of the MAFA and to eliminate the influence of constant multipath biases on the time-average model in static mode, the kinematic multi-epoch MAFA (kinematic ME-MAFA) algorithm is proposed. A heuristic method for predicting the ‘float position’ corresponding to every Voronoi cell of the next epoch, making use of Doppler-based velocity information, is implemented to improve the computational efficiency. If the success rate is very close to 1, it is possible to guarantee reliable centimeter-level accuracy positioning without further ambiguity validation. Therefore, a computing method of the success rate for the kinematic ME-MAFA is proposed. Both the numerical simulations and the kinematic experiment demonstrate the feasibility of the new AR algorithm according to its accuracy and reliability. The accuracy of the horizontal positioning solution is better than 1.7 centimeters in our pseudolite system.

## 1. Introduction

In recent decades, the landscape of global navigation satellite system (GNSS) technology has undergone far-reaching transformations to cater for both civilian and military needs, used throughout the national infrastructure for providing positioning, navigation, and timing services. However, the signal power of the GNSS is low, and hence, GNSS service may be limited or even not be available in GNSS-challenged environments where there is poor satellite geometry or signal blockage, such as indoors, in urban canyons, in mining pits, in tunnels, and underground, etc. A number of researchers have claimed that pseudolites are an emerging technology with the potential to extend the range of application of GNSSs, such as aircraft approach and landing [1], deformation monitoring applications [2], Mars exploration [3], maritime application [4], open-cut mining [5], indoor positioning [6], and others [7,8].

With the availability of precise GNSS satellite orbit and clock products, precise point positioning (PPP) technology can provide global centimeter or even millimeter positioning accuracy with a single receiver. Fixed PPP with integer carrier-phase ambiguity resolution (AR) can shorten the convergence time and improve the positioning accuracy significantly compared to float PPP [9]. The key to successful AR for PPP is to remove the fractional cycle bias (FCB) from the ambiguity. Some single-differenced (SD) FCBs estimation method and undifferenced FCBs estimation method are developed for recovering the integer property of the ambiguity parameter [10,11]. Learning from the idea of the GNSS PPP, an FCB correction method is proposed to recover the integer nature of single-difference ambiguities for the synchronized pseudolite system, and the experiment demonstrates the capability of real-time single-point positioning with centimeter or even millimeter positioning accuracy [12].

In the synchronized pseudolite system, centimeter-to-millimeter-level kinematic solutions can be achievable from the single-point positioning technique using carrier phase measurement. The primary task of carrier phase positioning is integer ambiguity fixing. Once the integer ambiguities are fixed correctly, the carrier-phase measurements are turned into very precise range measurements. As the pseudolite system is usually operated in the dense multipath environment, code measurements experience severe multipath effect, despite the use of advanced multipath-mitigation techniques. Phase-only processing is used to resolve the integer ambiguities for discarding the biasing effects that the code multipath brings [13].

A number of AR techniques are proposed for the pseudolite systems. The ‘known point initialization’ (KPI) directly calculate the integer ambiguities on a precisely surveyed point, but obviously is not convenient for practical kinematic applications [14]. To resolve the ambiguities, ‘on-the-fly’ (OTF) is preferred by most researchers, several OTF-AR techniques have been developed. Some OTF-AR techniques based on the least-squares method or the unscented Kalman filter are proposed for estimating the float ambiguities. Besides, some AR methods based on the conventional least-squares ambiguity decorrelation adjustment (LAMBDA) method for are proposed in [15,16].

Continuous carrier phase measurement at enough epochs should be observed to obtain an accurate float solution for the conventional LAMBDA method [17]. However, it is assumed that there is no cycle slip and the carrier phase integer ambiguities remain constant during the OTF-AR procedure in these papers [15,16]. When cycle slips occur, some bias will be propagated into the float solution, finally resulting in wrong integer solution estimated by the LAMBDA algorithm [18].

Another category of OTF AR algorithms is the search techniques in the coordinate domain, including the ambiguity function method (AFM) and the modified ambiguity function approach (MAFA), using only the fractional value of the carrier phase measurement, which are insensitive to the cycle slips [19].

There are several research studies based on the AFM method for the pseudolite application. An improved particle swarm algorithm to increase the search efficiency of single-epoch AFM is proposed, but the experiments proved that it requires an initial approximate coordinate precision better than 0.2 m to achieve centimeter-level precision positioning [20]. The MAFA is a few hundred times faster than the AFM, and can obviously reduce computational load [21]. However, the single-epoch MAFA model is too weak to resolve the integer ambiguity, hence more observations are required to improve model strength. Furthermore, due to the static nature of pseudolite transceivers, the multipath biases of the carrier phase measurement in the user receiver appear to be constant in static scenarios. If the ambiguities are biased, the probability of correct resolution will decrease rapidly [22]. In kinematic mode, however, the multipath biases vary randomly with the surrounding obstructs [23,24].

The MAFA methods are all based on a single-epoch or multi-epoch carrier phase measurements in the static GNSS-RTK mode [19,21,25,26]. Considering the current research status, the motivation for this paper is to extend the original MAFA to the kinematic mode. We propose a new algorithm, namely the kinematic multi-epoch MAFA (kinematic ME-MAFA), to improve the model strength and thus can increase the success rate of MAFA ambiguity estimation effectively, compared with the original MAFA methods in the static mode. The Doppler measurement is used to help resolve integer ambiguity for GNSS precise positioning applications in some research. Doppler-smoothed pseudorange is used for improving the fix rate of the single-epoch AR [27,28]. Besides, velocity from Doppler measurements is used to help single-epoch determination of carrier-phase integer ambiguities in the conventional LAMBDA method [29]; However, its single-epoch ‘float solution’ must be calculated by updating an accurate previous position regarded as the result of correct ambiguities. In this paper, a heuristic method making use of Doppler-based velocity information is proposed for improving the computational efficiency of the kinematic ME-MAFA, predicting the proposed ‘float solution’, corresponding to every MAFA Voronoi cell of the following multi-epoch without the need of an accurate priori position. Reliable centimeter-level accuracy positioning can only be guaranteed if the carrier phase integer ambiguities are correctly fixed. If the success rate is very close to 1, it is possible to rely on the integer solution without further validation. Therefore, a computing method of the success rate for the kinematic ME-MAFA is proposed.

## 2. Pseudolite Measurement Model

The signal tracking operation performed by the pseudolite receiver provides three types of instantaneous measurements, e.g., pseudorange, carrier phase, and Doppler shift. Taking account of the severe multipath effect on the pseudorange measurement, modeling and measurement error of the pseudolite carrier phase and Doppler shift are only presented.

The carrier phase measurement of the user corresponding to pseudolite *i* is represented as $\phi^{i}$ in unit of cycle:

(1) $$\phi^{i}=\lambda^{-1}\left(\rho^{i}+c\left(T^{i}+\delta t_{u}-\delta t^{i}\right)\right)+N^{i}+\varepsilon_{\phi }^{i},$$

where λ is the carrier wave length (in meters); $\rho^{i}$ is the geometric range between the antenna phase center of the *i*th pseudolite and that of the receiver; *c* is the speed of light; $\delta t_{u}$ and $\delta t^{i}$ are the clock offsets of the receiver and the pseudolite, respectively; $T^{i}$ is the tropospheric delay, which can be calculated using an appropriate model [30]; $N^{i}$ is the carrier phase integer ambiguity; $\varepsilon_{\phi }^{i}$ is the measurement noise, which may include residual tropospheric errors, multipath errors, receiver noise, etc.

Single-differenced operation is implemented to remove the receiver clock offset, the SD measurement between the *i*th pseudolite and the *j*th one is expressed as:

(2) $$\Delta \phi^{ij}=\phi^{i}-\phi^{j}=\lambda^{-1}\Delta \rho^{ij}+N^{ij}+\varepsilon_{\phi }^{ij}$$

where $\Delta \rho^{i}=\rho^{i}-\rho^{j}$, $N^{ij}=N^{i}-N^{j}$, $\varepsilon_{\phi }^{ij}=\varepsilon_{\phi }^{i}-\varepsilon_{\phi }^{j}$.

As nuisance fractional cycle bias parameter owing to the clock offset between two pseudolites $\delta t^{ij}=\delta t^{i}-\delta t^{j}$ can be eliminated by the synchronized pseudolite system in the transmitter end, SD carrier phase ambiguity is retrieved to be integer in nature [12].

The Doppler measurement, although less precise compared with the carrier phase measurement, has the advantage of being immune to cycle slips [31]. In addition, the noise and the multipath of delta range derived from the Doppler measurement are significantly smaller than that of code measurements.

The raw Doppler measurement $D^{i}$ corresponding to pseudolite *i*, in units of Hz can be simply modeled as:

(3) $$\lambda D^{i}={\dot{\rho }}^{i}+c\left(\dot{\delta }t_{u}-\dot{\delta }t^{i}\right)+\varepsilon_{D}^{i},$$

where

(4) $${\dot{\rho }}^{i}=-l^{i}v,$$

${\dot{\rho }}^{i}$ is range rate; $l^{i}$ is the line-of sight unit vector between the receiver and the pseudolite; v is the instantaneous velocity of user; $\dot{\delta }t_{u}$ and $\dot{\delta }t^{i}$ are the clock drifts of the receiver and the pseudolite respectively; $\varepsilon_{D}^{i}$ is Doppler measurement noise; As the sample interval of the Doppler measurement is generally no longer than one second, small gradient of the tropospheric delay is ignored [32].

## 3. AR Methodology

The proposed algorithm in this paper is based on the MAFA AR method. MAFA is an ambiguity estimation approach, determining the right integer ambiguity based on the search in the coordinate domain.

### 3.1. Single-Epoch MAFA

The single epoch SD carrier phase measurement can be represented by the following simplified equation:

(5) $$\Phi +e=\frac{1}{\lambda }\rho \left(b\right)+a,$$

where Φ is the SD carrier phase measurement vector; b is the unknown coordinate vector of user receiver; ρ(b) is the SD geometrical range vector; a is the unknown SD carrier phase ambiguity vector; e is measurement error vector of SD carrier phase measurement.

Typically, the noise of carrier phase measurement is about 0.01~0.04 cycle [33,34], much lower than a half cycle. Taking account of the integer nature of SD ambiguities of synchronized pseudolite system, the SD ambiguity vector then has the following form:

(6) $$a=round\left(\Phi -\frac{1}{\lambda }\rho \left(b\right)\right),$$

where round(⋅) is a function of rounding to the nearest integer value.

Substituting Equation (6) into Equation (5), the error equations can be shown as:

(7) $$e=\left(\Phi -\frac{1}{\lambda }\rho \left(b\right)\right)-round\left(\Phi -\frac{1}{\lambda }\rho \left(b\right)\right)$$

After a Taylor series expansion, a linearized form of the error equations can be rewritten as:

(8) e=δ−BΔb,

with:

(9) $$\delta ={\left[\begin{array}{cccc} \delta_{1} & \delta_{2} & \cdots & \delta_{n} \end{array}\right]}^{T},$$

(10) $$\delta_{n}=\left(\Phi -\frac{1}{\lambda }\rho \left(b_{0}\right)\right)-round\left(\Phi -\frac{1}{\lambda }\rho \left(b_{0}\right)\right),$$

(11) $$\Delta X={\left[\begin{array}{ccc} dx & dy & dz \end{array}\right]}^{T},$$

(12) $$B=\left[\begin{array}{ccc} \frac{\partial \rho_{1}}{\partial x} & \frac{\partial \rho_{1}}{\partial y} & \frac{\partial \rho_{1}}{\partial z}\\ \frac{\partial \rho_{2}}{\partial x} & \frac{\partial \rho_{2}}{\partial y} & \frac{\partial \rho_{3}}{\partial z}\\ \vdots & \vdots & \vdots \\ \frac{\partial \rho_{2}}{\partial x} & \frac{\partial \rho_{2}}{\partial y} & \frac{\partial \rho_{2}}{\partial z} \end{array}\right],$$

where $b_{0}={\left[\begin{array}{ccc} x_{0} & y_{0} & z_{0} \end{array}\right]}^{T}$ is a priori coordinate vector. Additionally, *n* is the number of SD observations per epoch. B is the (*n* × 3) design matrix. δ is the (*n* × 1) residual vector.

Equation (8) is solved with nonlinear least squares (LS) adjustment method:

(13) $$\arg\;\underset{X}{\min}\left(\varepsilon_{\mathrm{WSSE}}=\overset{⌣}{e}Q_{\Phi }^{-1}\overset{⌣}{e}\right),$$

where $\overset{⌣}{e}$ is a LS estimate of measurement error vector e in Equation (8). $\varepsilon_{\mathrm{WSSE}}$ is the weighted sum squared error of the LS adjustment. If $\sigma_{0}$ is the variance of carrier phase measurement, $Q_{\Phi }=E\left\{ee^{T}\right\}=\sigma_{0}^{2}D$ is the variance–covariance matrix of SD measurement vector Φ, with:

(14) $$D=\left[\begin{array}{cccc} 2 & 1 & \cdots & 1\\ 1 & 2 & \cdots & 1\\ \vdots & \vdots & \ddots & \vdots \\ 1 & 1 & \cdots & 2 \end{array}\right]$$

The solution of the above nonlinear model must be Newton-iterated to obtain the convergent result. Then, the LS coordinate increment solution $\Delta \overset{⌣}{b}$ is obtained by the following equation:

(15) $$\Delta \overset{⌣}{b}=\frac{1}{\lambda }{\left(A^{T}Q_{\Phi }^{-1}A\right)}^{-1}A^{T}Q_{\Phi }^{-1}\delta .$$

The fix coordinate solution $\overset{⌣}{b}$ of MAFA can be then obtained by:

(16) $$\overset{⌣}{b}=b_{0}+\Delta \overset{⌣}{b}.$$

Although the ambiguity parameters are not presented in the model in (8), substituting LS coordinate solution $\overset{⌣}{b}$ into Equation (6), the final constrained SD ambiguity estimate can be obtained by:

(17) $$\overset{⌣}{a}=round\left(\Phi -\frac{1}{\lambda }\rho \left(\overset{⌣}{b}\right)\right).$$

The pull-in regions in the ambiguity domain from the Teunissen theory are sometimes referred to also as Voronoi cells [35]. Similar to the Voronoi cell in the ambiguity domain, there is a defined Voronoi cell in the coordinate domain. However, the Voronoi cells in MAFA are different. A so-called good Voronoi cell in the coordinate domain is defined in [25]. A good Voronoi cell is a graphical interpretation of Equation (6) in the coordinate domain. For obtaining the correct ambiguities in Equation (6), the following condition must be met:

(18) $$\left|e-e_{\rho^{0}}/\lambda \right|<0.5,$$

where $e_{\rho^{0}}$ is the vector of the computed SD geometrical distance errors corresponding to the a priori coordinate $b_{0}$. Condition (18) is a necessary and sufficient condition to obtain a correct solution in MAFA.

**Figure 1:**
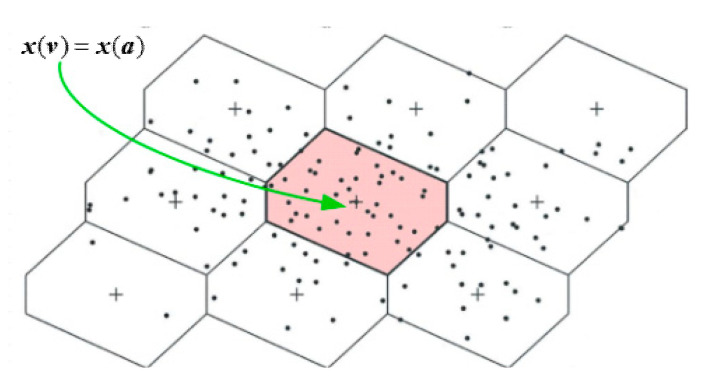
Illustration of the Voronoi cell in 2D.

Figure 1 presents an example of a few Voronoi cells and some cloud of float positions in 2D space. The good Voronoi cell is located in the center. The shape and the size of the Voronoi cell depend on the geometry configuration and the carrier wavelength, respectively. These cells in the coordinate domain can be empirically generated based on Equation (6) from a dense set of points.

The MAFA method can give correct solutions only if an a priori position falls into the good Voronoi cell. If the priori position is accurate enough to satisfy Condition (18), then it can be pulled into the good Voronoi cell constructed for the true position. Otherwise, it will be pulled to a bad cell in the coordinate domain that corresponds to the wrong integer ambiguities, in accordance with Equation (6) [21].

The optimization problem in MAFA has the form of (8) and (13), in which the objective function is the weighted sum squared error $\varepsilon_{\mathrm{WSSE}}$ of the LS adjustment. The search in the coordinate domain grid by grid is needed to obtain the global minimum, which corresponds to the correct fix-solution of the objective function. However, the objective function is a multi-valley function. With meter-level accuracy of approximate coordinate, using only one epoch phase observation or multi-epoch observations with the time-average operation in static mode makes the objective function noisy or biased, resulting in low success rate of getting the fix solution.

### 3.2. Kinematic ME-MAFA

**Figure 2:**
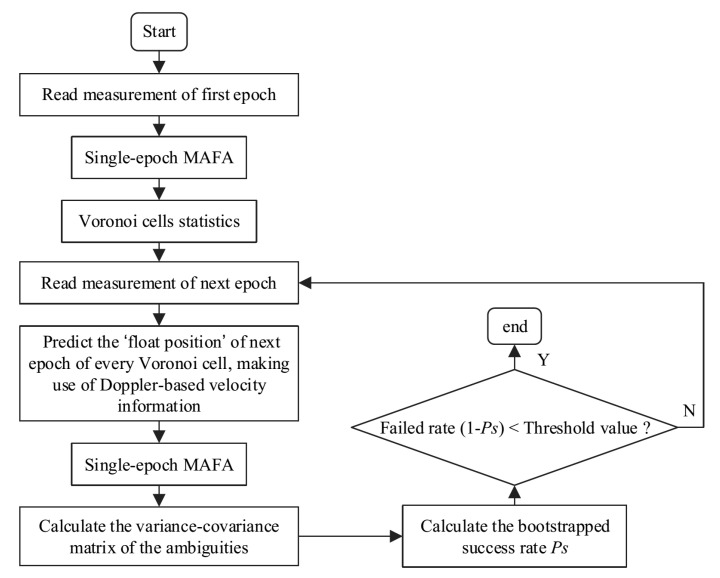
Procedure of the proposed kinematic ME-MAFA algorithm.

The success rate of MAFA using single epoch or multi-epoch observations with simple time-average operation in static mode is usually very low, which restricts MAFA application. Therefore, the kinematic ME-MAFA is proposed to improve the model strength and thus can increase the success rate of MAFA ambiguity estimation effectively. The algorithm of the kinematic ME MAFA algorithm is shown in Figure 2.

#### 3.2.1. Ambiguity estimation of kinematic ME-MAFA

For consecutive *M*-epochs carrier phase observations, the ambiguity vector shall have the form [21]:

(19) $$a_{\mathrm{ME}}=\left[\begin{array}{ccccc} a_{1} & \cdots & a_{m} & \cdots & a_{M} \end{array}\right],\;m=1,2,\cdots ,M.$$

In addition, the MAFA is immune to cycle slips even if processing multiple-epochs observations, which has been proven in [19].

The linearized error equations of *M* epochs in kinematic mode can be simply written as:

(20) $$e_{\mathrm{ME}}=\delta_{\mathrm{ME}}-B_{\mathrm{ME}}\Delta b_{\mathrm{ME}},$$

with:

(21) $$e_{\mathrm{ME}}={\left[\begin{array}{cccc} e_{1} & e_{2} & \cdots & e_{M} \end{array}\right]}^{T},$$

(22) $$\delta_{\mathrm{ME}}={\left[\begin{array}{cccc} \delta_{1} & \delta_{2} & \cdots & \delta_{M} \end{array}\right]}^{T},$$

(23) $$\Delta b_{\mathrm{ME}}={\left[\begin{array}{cccc} \Delta b_{1} & \Delta b_{2} & \cdots & \Delta b_{M} \end{array}\right]}^{T},$$

(24) $$B_{\mathrm{ME}}=\left[\begin{array}{cccc} B_{1} & & & \\ & B_{2} & & \\ & & \ddots & \\ & & & B_{M} \end{array}\right].$$

Thanks to the absence of undetermined ambiguity vector $a_{\mathrm{ME}}$ in the linearized MAFA model, there is no any derivative sub-matrix corresponding to the integer ambiguity parameters in the design matrix $B_{\mathrm{ME}}$, compared with the conventional LS method for estimating the float ambiguities. Thus, the batch-proposing all epoch observations together popular in the conventional LS method can be avoided, which is computing-time consuming due to the large size of observation matrix in Equation (20).

For proposed kinematic ME-MAFA, every single epoch observation shall be input to the model in Equation (8) individually. Additionally, the objective function of the kinematic ME-MAFA has the following form:

(25) $$\varepsilon_{\mathrm{WSSE}\text{-}\mathrm{ME}}=\frac{1}{M}\sum_{i=1}^{M}\left(\varepsilon_{\mathrm{WSSE}}(m)\right),\;m=1,2,\cdots ,M,$$

with $\varepsilon_{\mathrm{WSSE}}(m)={\overset{⌣}{e}}_{m}Q_{\Phi }^{-1}{\overset{⌣}{e}}_{m}$; *M* is the number of the observation epochs. The objective function of kinematic ME-MAFA is the sum of objective function of every single epoch MAFA straightforward.

Apparently, a trivial search procedure epoch by epoch can be implemented to find the correct solution. However, if the priori coordinate of every epoch is poor and the needed epochs for high success rate of AR is large, independent search of multi-epochs have so many search grids, that computation time may be too long for (near) real-time applications.

Taking account of the long computation time of independent search of multi-epochs, a heuristic method making use of user velocity information is proposed to improve the computational efficiency. Firstly, the search grid by grid is conducted for the first epoch, using the single-epoch MAFA model in (8). The subsequent epochs after the first epoch will take advantage of the Doppler-based velocity.

The time-differenced carrier phase is sensitive to the cycle slip, thus the raw Doppler measurement is widely used to estimate the user velocity [36]. The Doppler measurement is also subject to the frequency bias between the pseudolite oscillator and the receiver oscillator, thus the unknowns in the Doppler model are the receiver antenna phase-center velocity vector $v={\left[\begin{array}{ccc} v_{x} & v_{y} & v_{z} \end{array}\right]}^{T}$ and the clock drift $\dot{\delta }t=\dot{\delta }t_{u}-\dot{\delta }t^{i}$. The three-dimensional velocity of user receiver is solved with nonlinear least squares (LS) adjustment method:

(26) $$\left[\begin{array}{c} \Delta v\\ \Delta \dot{\delta }t \end{array}\right]={\left(H^{T}Q_{D}^{-1}H\right)}^{-1}A^{T}Q_{D}^{-1}\Delta D,$$

where the (*n* + 1) dimensional H design matrix of computing the velocity is similar to the one used to compute the position starting from the pseudorange measurement. Additionally, *n* + 1 is the number of visible pseudolites. $Q_{D}$ is the variance–covariance matrix of the Doppler measurement.

The ‘float solution’ of next epoch can be predicted by dead-reckoning from a previous fix position with average Doppler-based velocity, given by the following prediction equation:

(27) $$\hat{b}(m+1)=\overset{⌣}{b}(m)+\bar{v}(m)\cdot T_{s},$$

where $T_{s}$ is the sampling interval of carrier phase observation. $\bar{v}(m)$ is the average Doppler-based velocity between two epochs. $\overset{⌣}{b}(m)$ is the fix coordinate of previous epoch calculated by the single-epoch MAFA model in (15) and (16).

**Figure 3:**
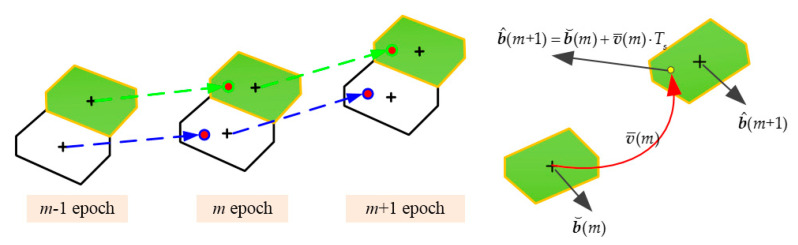
Illustration of the kinematic ME-MAFA in 2D.

Doppler-based velocity is reliable, and can reach an accuracy of mm/s to cm/s [37]. If the dynamics of the user is not high, so the predicted ‘float solution’ can be dropped into the Voronoi cell of the fix coordinate of the next epoch. Then, the predicted ‘float solution’, as the priori coordinate, is inputted in the single-epoch MAFA model for solving the fix solution of next epoch, as illustrated in Figure 3.

A few grids located in the same Voronoi cell generally are all pulled into the center of this cell as shown in Figure 1, thus the number of the candidates for all of the subsequent epochs shall be decreased naturally after the search of the first epoch. This is the reason that the proposed kinematic multi-epoch MAFA, making use of Doppler-based velocity information, can reduce the computational burden for real-time pseudolite precise positioning.

#### 3.2.2. Error Analysis of Float Solution

The accuracy of the proposed ‘float solution’ depends on the accuracy of the Doppler-based velocity. Raw Doppler measurements can be directly obtained by the output of the phase lock loop (PLL) filter. The Doppler tracking jitter in a GNSS receiver is given by:

(28) $$\sigma_{D}=\frac{1}{2\pi T_{coh}}\sqrt{\frac{B_{L}}{C/N_{0}}},$$

where $T_{coh}$ is coherent time (s), which is equivalent to the loop update interval in general; $B_{L}$ is the loop bandwith (Hz); *C*/*N*_0_ (dB-Hz) is the carrier to noise ratio of received navigation signal.

Time division multiple access (TDMA) is adopted to overcome the well-known near-far problem in the pseudolite system. Each pseudolite transmitter is identified by a unique transmission slot. For the TDMA scheme of the pseudolite system, the Doppler tracking jitter due to thermal noise in a pseudolte receiver is given by:

(29) $$\sigma_{D}=\frac{PDC}{2\pi T_{coh}}\sqrt{\frac{B_{L}}{C/N_{0}\cdot PDC}},$$

where PDC is the pulse duty cycle (range from 0 to 100%) of the pseudolite signal. The loop update interval is equivalent to $T_{coh}/PDC$. The coherent time $T_{coh}$ is determined by the pulse time-width.

**Figure 4:**
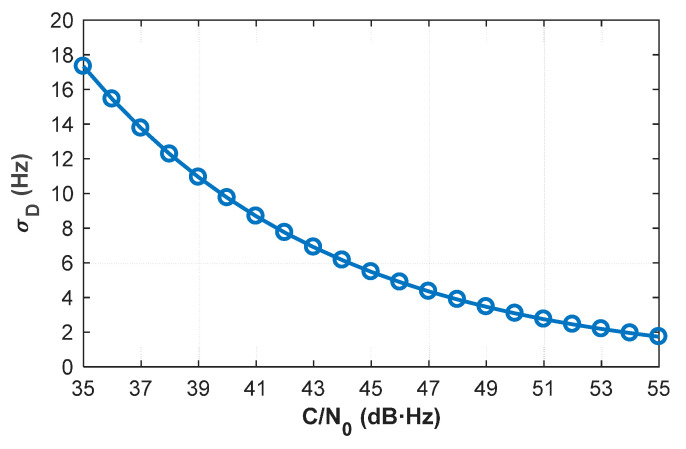
Theoretical Doppler jitters as a function of different *C*/*N*_0_ values.

The coherent time $T_{coh}$, the pulse duty cycle PDC, and the loop bandwidth $B_{L}$ of the designed receiver in our pseudolite system are set to 0.2 ms, 10%, and 15 Hz, respectively. The theoretical Doppler jitters as function of the *C*/*N*_0_ are shown in Figure 4.

The random noise of raw Doppler can be lowered by the time-average processing. The average Doppler observation is written as:

(30) $$\bar{D}(m)=\frac{1}{K}\sum_{k=1}^{K}D_{k},$$

where *K* is the number of raw Doppler observations in a sampling interval of the carrier phase observation. In addition, its noise can be derived according to the law of variance propagation as:

(31) $$\sigma_{\bar{D}}=\frac{\sigma_{D}}{K}.$$

The raw Doppler measurement from the PLL is updated at a high rate up to 500 Hz in our pseudolite system. If the sampling interval is set to 0.2 ms, then K is equal to 100. When *C*/*N*_0_≥35 dB-Hz, according to Equation (31), the standard deviation of average Doppler is lower than 0.18 Hz.

The average velocity derived from the smoothed Doppler measurement can be approximated as an instantaneous velocity for such a small sampling interval [38]. For the BeiDou B1 signal (λ = 19.2 cm), if the dilution of precision is no higher than 2.9, the average velocity can still reach the accuracy of centimeter per second. Under the above circumstance, according to Equation (27), the standard deviation of estimated ‘float solution’ is better than 2.5 cm.

#### 3.2.3. Computing Success Rate of Kinematic ME-MAFA

A very high positioning performance can only be guaranteed if the estimated integer ambiguities are correct. If the success rate is very close to 1, it is possible to rely on the integer solution without further validation [39]. In practice, a user does not want to use the integer solution if the failure rate is too large.

Therefore, it is very important to assess the success rate. MAFA can obtain exactly the same SR values as conventional integer least squares (ILS) [21]. The success rate of ILS cannot be evaluated exactly, but the bootstrapped success rate is known to be a tight lower-bound. The bootstrapped success rate can be calculated as [40]:

(32) $$P_{s}=\prod_{i=1}^{n}\left(2\Phi \left(\frac{1}{\sigma_{{\hat{z}}_{i|I}}}\right)-1\right),$$

(33) $$\Phi (x)=\frac{1}{\sqrt{2\pi }}\int_{-\infty }^{x}\exp\left(-\frac{1}{2}v^{2}\right)dv,$$

where $\sigma_{{\hat{z}}_{i|I}}$ is the conditional standard deviation after decorrelating Z-transformation of the variance-covariance matrix $Q_{\hat{a}\hat{a}}$ of the float ambiguities.

There are no float ambiguities solutions and their corresponding variance–covariance matrix in the proposed kinematic ME-MAFA. The corresponding variance-covariance matrix is estimated as:

(34) $$Q_{\hat{a}\hat{a}}={\left(A^{T}P_{yy}A-A^{T}P_{yy}B_{\mathrm{ME}}{\left(B^{T}P_{yy}B_{\mathrm{ME}}\right)}^{-1}B_{\mathrm{ME}}{}^{T}P_{yy}A\right)}^{-1},$$

with:

(35) $$A=\left[\begin{array}{cccc} I_{1} & & & \\ & I_{2} & & \\ & & \ddots & \\ & & & I_{M} \end{array}\right],$$

where $I_{1}=I_{2}\cdots =I_{M}$ is the *n*-dimensional identity matrix.

The design matrix $B_{\mathrm{ME}}$ is computed from the fix solution ${\overset{⌣}{b}}_{\mathrm{ME}}={\left[\begin{array}{cccc} {\overset{⌣}{b}}_{1} & {\overset{⌣}{b}}_{2} & \cdots & {\overset{⌣}{b}}_{M} \end{array}\right]}^{T}$ after the minimum search of the objective function in Equation (25).

## 4. Numerical Simulations

**Figure 5:**
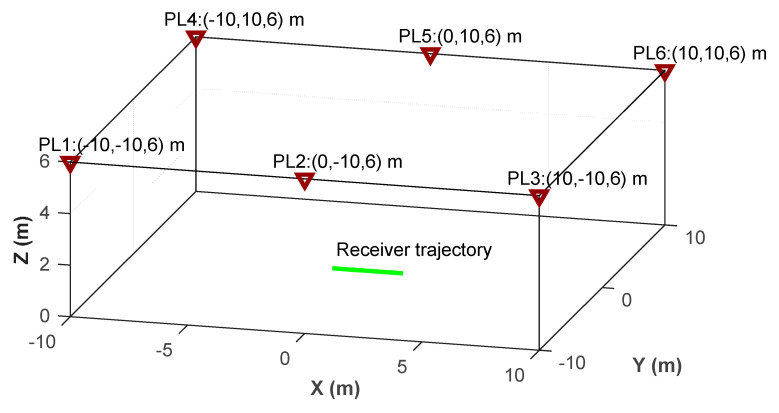
Sketch of the pseudolite system in the simulation.

The performance of the kinematic ME-MAFA AR method will be investigated under a variety of functional and stochastic models, to make a guideline for the real-world experiment in the next section and for the future applications. The coordinates of six pseudolite layouts in a defined Cartesian coordinate system are shown in Figure 5. It is assumed that the receiver moves along the x-axis in the center of the coverage region symmetrically (Y = 0, Z = 0). The speed of the receiver is assumed to be 1 m/s. The carrier frequency of BeiDou B1 signal (λ = 19.2 cm) is used. The simulated integer ambiguities are the round results of the theoretical geometric distances corresponding to the first epoch divided by the carrier length. The SD carrier phase measurement is the result of theoretical SD geometric distances of all epochs minus the SD integer ambiguities. The differencing is conducted against the PL1. The carrier phase data and the raw Doppler data are generated with sampling rates of 5 and 500 Hz, respectively. The pseudolite transceivers and the user receiver of the pseudolite system are designed by ourselves. Thus, the sample rate of the raw measurement can be changed according to our need. The updated interval of the signal tracking loops is 2 ms in our receiver, so the sample rate of the Doppler measurement can be as high as 500 Hz.

**Figure 6:**
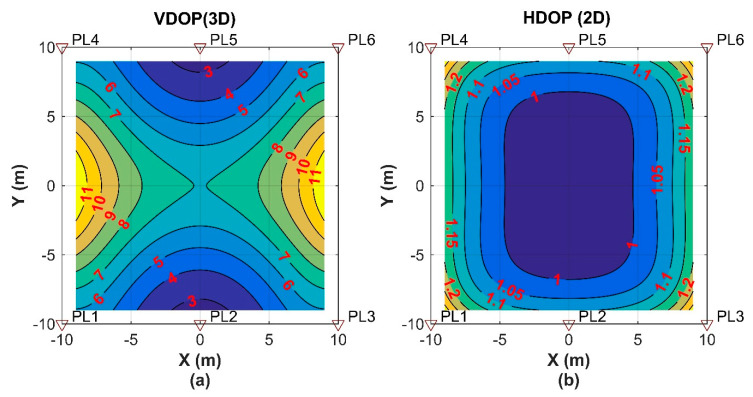
VDOP (vertical dilution of precision) and HDOP (horizontal dilution of precision) in 2D mode of the pseudolite layout. (**a**): VDOP for 3D positioning; (**b**): HDOP for 2D positioning implemented in this paper.

The average vertical dilution of precision (VDOP) for three-dimensional (3D) positioning is 6.59. The VDOP of the pesudolite system is commonly higher compared with the corresponding term of GNSS. Integration with GNSS is adopted to improve the poor vertical geometry of the pseudolite system in [41]. The layout in the real-world experiment is similar to the term in the simulations. Therefore, for the standalone pseudolite system, two-dimensional (2D) positioning is implemented in the following simulations and the real-world experiment. The horizontal dilution of precision (HDOP) for 2D positioning in the whole coverage area is mostly smaller than 1.15. The average HDOP for 2D positioning is 1.05 for this simulated layout, and the HDOPs in the whole coverage area are mostly smaller than 1.15. The VDOP for 3D positioning and HDOP for 2D positioning implemented in this paper are shown in Figure 6.

The noise of simulated measurements is assumed as Gaussian white noise. For each Monte-Carlo simulation scenario, a set of 10^4^ or 10^5^ noise vectors are generated. Then, each simulation for the different measurement noise and for the different geometry change was repeated. The density of the grid of candidates or the searching step is set at half of the carrier wavelength in all simulation scenarios, according to [26].

### 4.1. Influences of Measurement Noise and Search Region

**Figure 7:**
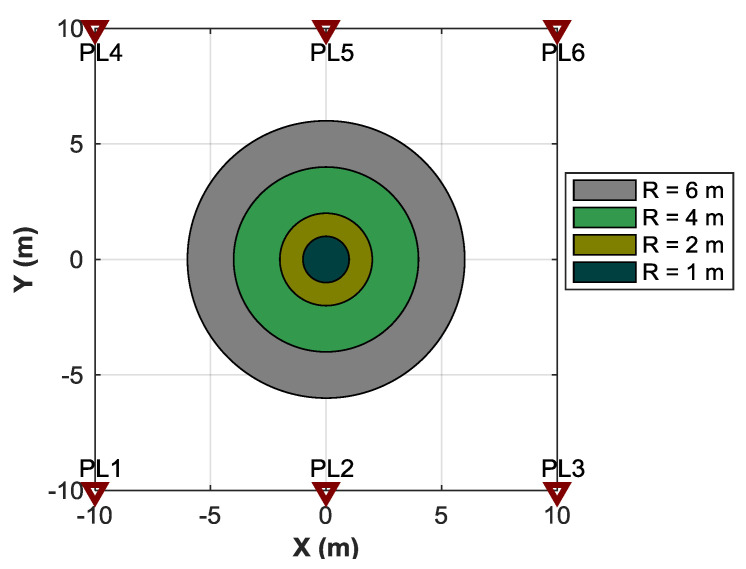
Illustration of the search regions with different radius.

The first test is intended to investigate the success rates of the kinematic ME-MAFA ambiguity estimation under different measurement noise and different search region. The success rate is defined as the percentage of the integer estimation equal to the correct integer vector [39]. The moving distance of the receiver is 2 m, thus observations of 11 epochs are generated. The measurement noise of the undifferenced carrier phase measurement is assumed to range from 0.03 to 0.05 cycle. The standard deviation of the Doppler measurement is 20 Hz. The circle radius (R) of the search region corresponding to the prior coordinate of the first epoch is assumed to range from 1 to 6 m. Four search regions with different radius are shown in Figure 7.

Table 1 shows the simulation-based success rates as function of the phase noise levels and the circle radius of the search region. For each Monte-Carlo simulation scenario, a set of 10^4^ noise vectors are generated. The results show that the success rates depend on the precision of the carrier phase observation. When the noise level is lower, the success rate is higher. More importantly, large size of the search region has no influence on the success rate of MAFA, which is also verified by some simulations in [21].

Doppler-based velocity information is proposed to improve the computational efficiency. A few search grids may be pulled into the center of the same Voronoi cell. Thus, after the search corresponding to the first epoch, the number of Voronoi cells is smaller than the original grids in the search circle of the first epoch. Table 2 shows the original grids in the search circle and the value of the number of the Voronoi cells. For the search radius ranging 1 to 6 m, the candidates are decreased by 24.63%, 24.39%, 25.36%, and 26.24% respectively. Therefore, the computation time of the kinematic ME-MAFA using the Doppler-based velocity information can be shortened by about a quarter in this simulation.

### 4.2. Influence of the Cycle Slip

To investigate the AR performance of the proposed algorithm with the unexpected cycle slip, another simulation is conducted. Then, one-cycle slip is inserted into the simulated carrier phase measurement of PL5 at the 6th epoch. The search radius is set to 1 m. For all of the following Monte-Carlo simulation scenarios, a set of 10^5^ noise vectors are generated. Success rates with the cycle slip are summarized in Table 3, which is exactly identical to the corresponding terms without the cycle slip. The kinematic ME-MAFA is verified to be resistant to the cycle slips, similar to conventional AFM and MAFA. When there are no any cycle slips, LAMBDA obtains almost the same success rate values as the proposed kinematic ME-MAFA. However, when there is a cycle slip, our method clearly provides much better results than the conventional LAMBDA method. In addition, the MAFA has the weakness in the computation efficiency, and the computation time in MAFA is ten or so times longer than in the LAMBDA [21].

### 4.3. Influence of Geometry Change

The model of kinematic ME-MAFA is a geometry-based model. For the pseudolite geometry-based model, the user-pseudolite geometry change is a critical factor in terms of improving OTF-AR performance. Due to the static nature of pseudolite transceivers, geometry change can only be generated with the movement of the user receiver [42].

To investigate the influence of the geometry change on the AR performance of the kinematic ME-MAFA, the third simulation is conducted. The moving distance of the user receiver ranges from 1 to 4 m. The noise level is set to 0.04 cycle. The kinematic ME-MAFA is a multi-epoch AR method. The number of epochs using for AR increases with the moving distance. The statistical results of the simulation in the fourth column of Table 4 show that the success rate is improved significantly with the increment of the moving distance of the user receiver, owing to more and more epochs used to fix the integer ambiguities. When the moving distance is longer than 3 m, the success rate is higher than 99.99%. When the moving distance is 4 m, there is no any wrongly fixed solution. The theoretical lower bound based on the bootstrapped success rate and the theoretical upper bounds based on the ambiguity dilution of precision (ADOP) for the ILS success rate are also listed in Table 4. All the lower bounds and the upper bounds of the success rates are calculated by using the Ps-LAMBDA toolbox, and the designers indicate that for some cases, the ADOP-based upper bound often gives a too optimistic value compared to the actual success rate [39].

**Figure 8:**
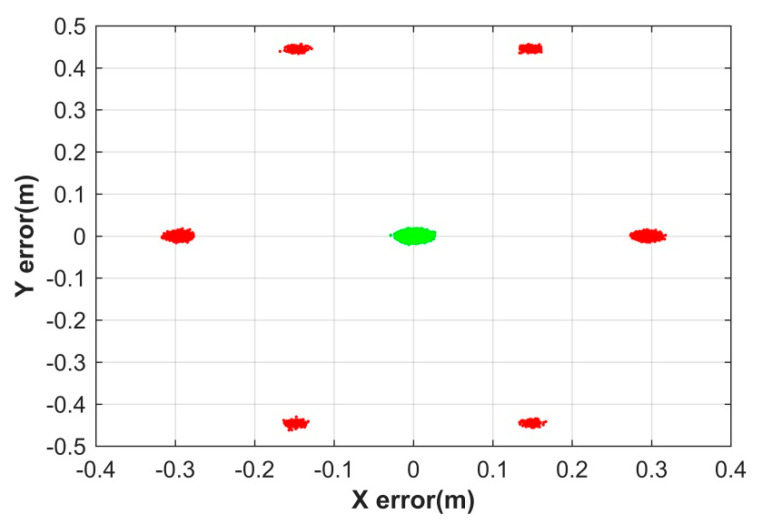
Scatterplot of positioning errors for the correct fix solution (green dots) and wrong fix solution (red dots).

As is illustrated in Figure 8, positioning errors of correctly fixed solution (green scatter) are centimeter-level; however, the large red scatter indicates that the success rate is not large enough (only 88.646%). As a result, some of wrongly fixed positions even cause decimeter-level positioning errors. This underlines that the fixed solution should only be used when the success rate is sufficiently high.

**Figure 9:**
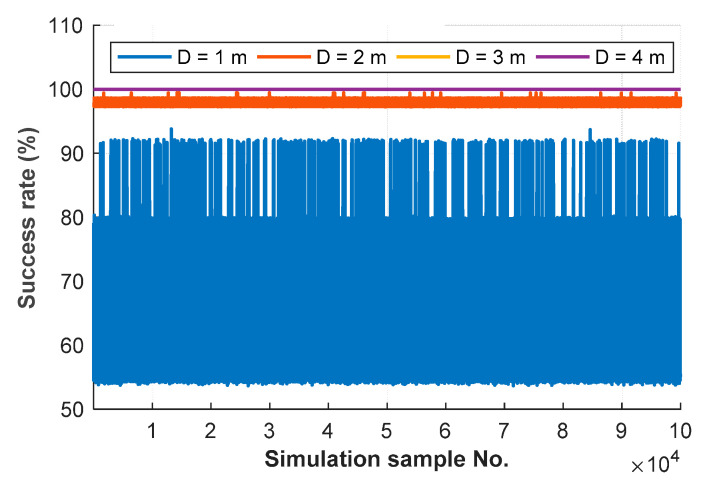
Illustration of computed lower-bound success rates of 10^5^ samples of five geometry configurations, according to Equation (32) and Equation (34).

Figure 9 shows computed lower-bound success rates of 10^5^ samples of four geometry configurations, according to Equations (32) and (34). For smaller geometry change, the theoretical lower-bound success rate is lower, as shown in the middle column of Table 4; and the computed lower-bound success rates are lower and their fluctuation is larger due to wrong fix solutions. For visualization purposes, the fail rate is shown in Table 5 instead of the success rate. For 4 m of moving distance without any wrongly fixed solution, the statistical fail rate is approximately equal to the theoretical success rate. Thus, for reliable AR without further validation in the real-world applications, the threshold value of the fail rate is set to 5 × 10^-9^, according to the guideline from this simulation.

## 5. Real-World Experiments

**Figure 10:**
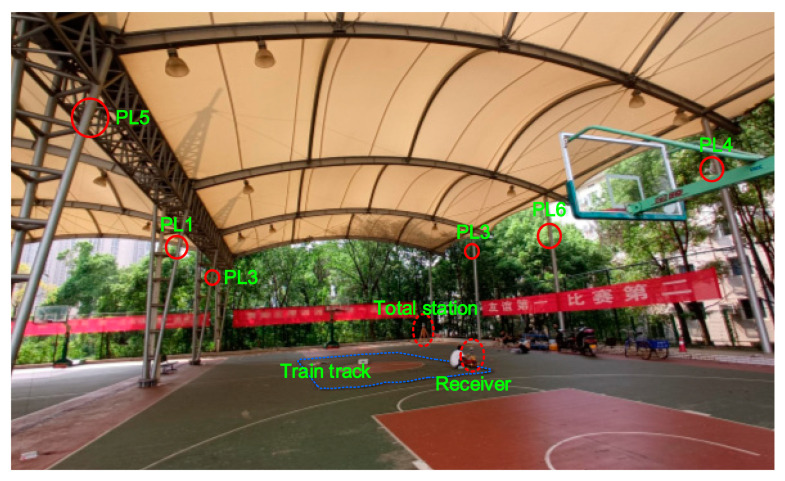
Six pseudolites and the user receiver in the test field.

To validate that the kinematic ME-MAFA is capable of producing reliable AR, the field test is conducted at a semi-enclosed basketball court in National University of Science and Technology on August 30, 2020. The experimental pseudolite system consists of six pseudolite (PL1~PL6) transceivers and a user receiver, as shown in Figure 10. Antennas of six pseudolies are mounted on the metal columns. PL1 was chosen as the master pseudolite and all other slave pseudolites are synchronized with it. Additionally, the PL1 is chosen as the reference pseudolite. The coordinates of each pseudolite transmitting antenna are listed in Table 6, determined by a total station setting at the margin of test field. The antenna of user receiver is installed on a toy train, moving along the train track at an approximate speed of 0.75 m/s. The 2D positioning experiments are conducted. The height of the receiver antenna is fixed at 0.471 m. During the circumnavigation, HDOP values are between 0.98 and 1.1.

**Figure 11:**
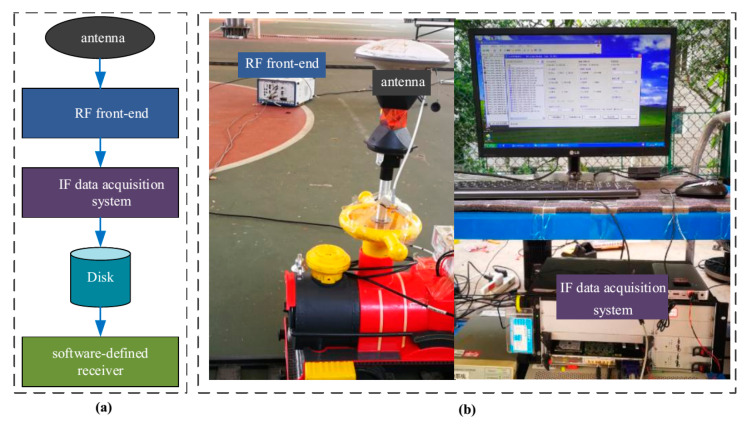
The architecture of the data collection system and hardware setup for the software receiver. (**a**): Architecture of the data collection system; (**b**): Hardware setup for the software receiver.

The radio-frequency (RF) signal from the antenna is fed to the RF front-end system, where the RF signal is down-converted to the intermediate frequency (IF) signal. Then, the IF signal is digitized at the data acquisition system. The measurements are obtained by processing the digitized IF samples using a software-defined receiver. The architecture of the IF data collection system and the photos of the hardware setup of the software receiver are shown in Figure 11.

**Figure 12:**
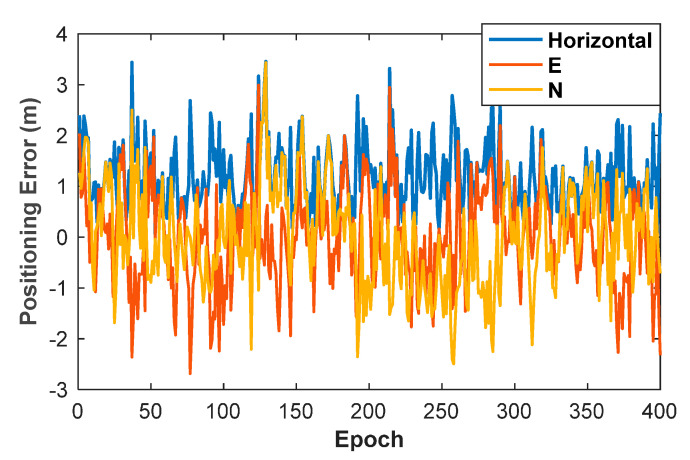
Positioning error of pseudorange measurement.

The priori position of the kinematic ME-MAFA is determined using the pseudorange measurement. In order to counter the pseudorange multipath in the terrestrial pseudolite system, the chip-rate is increased to 10.23 million chips per second. The pseudorange positioning errors are shown in Figure 12, and the maximum of horizontal positioning errors is 3.45 m. To achieve reliable AR, the search radius of the kinematic ME-MAFA is set to 4 m.

**Figure 13:**
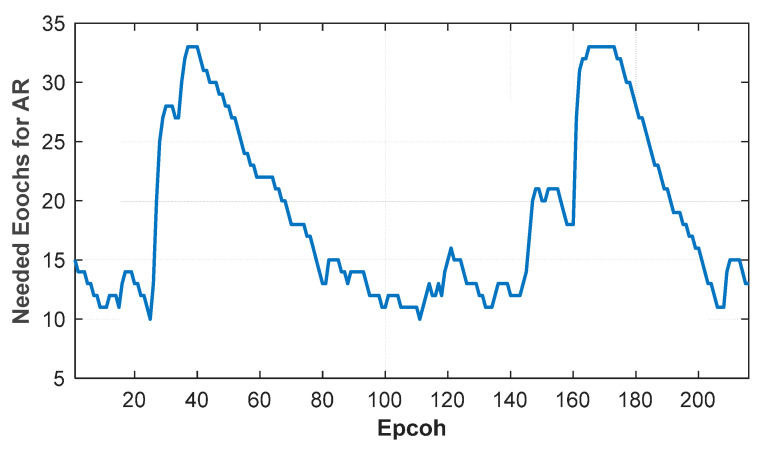
Epochs needed for reliable AR (ambiguity resolutions) during the round trip.

The kinematic ME-MAFA algorithm uses a variable number of epochs for a multi-epoch AR until reliable AR is achieved. During a round trip, the integer ambiguities of all test groups are estimated correctly, and the actual success rate is 100%. The numbers of epochs needed to meet the threshold value of the fail rate are shown in Figure 13. The maximum and the average of the number of epochs needed for AR are 33 and 19 (or 6.6 and 3.8 seconds of carrier phase observation), respectively. Due to the poor accuracy of the priori position, the number of search grids in the 4 m radius circle is 5449. After the search of the first epoch, the maximum and the average of the number of the Voronoi cells are decreased to 3710 and 3419. Therefore, the computation time of the kinematic ME-MAFA using the Doppler-based velocity information is shortened by about 37.2% on average. The average computation time in these searches is 26.1 seconds (MATLAB Windows environment on a 2.6 GHz Intel CPU).

To investigate the AR performance of the proposed algorithm with cycle slips, artificial cycle slips are inserted into the raw phase measurements. Table 7 summarizes the information about the data sets and the corresponding AR performances. The integer ambiguities are fixed correctly for all data sets regardless of epoch, pseudolite, and size of the cycle slip.

**Figure 14:**
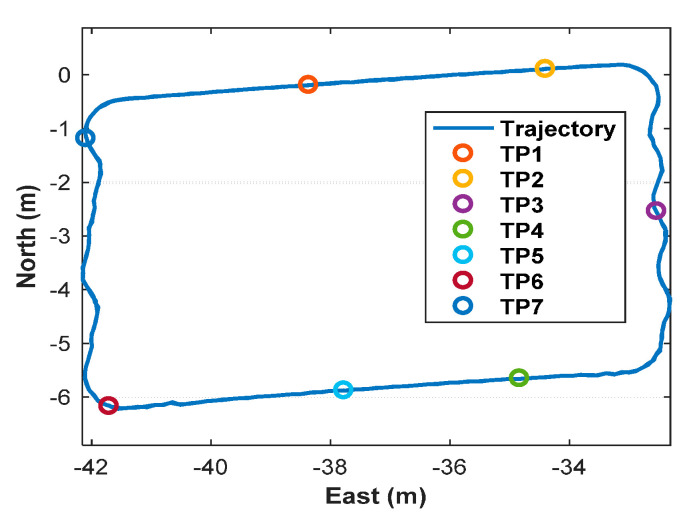
The estimated trajectory of the toy train.

In order to further judge the correctness of the fixed integer ambiguities, the estimated trajectory of the toy train is plotted in Figure 14. The trajectory is quite consistent during the circumnavigation visually. For precision and accuracy examination, the "stop-and-go" mode is employed in the following test, and the receiver occupied seven test points (TPs) for about 18~28 s. True coordinates of seven TPs are determined by the total station.

**Figure 15:**
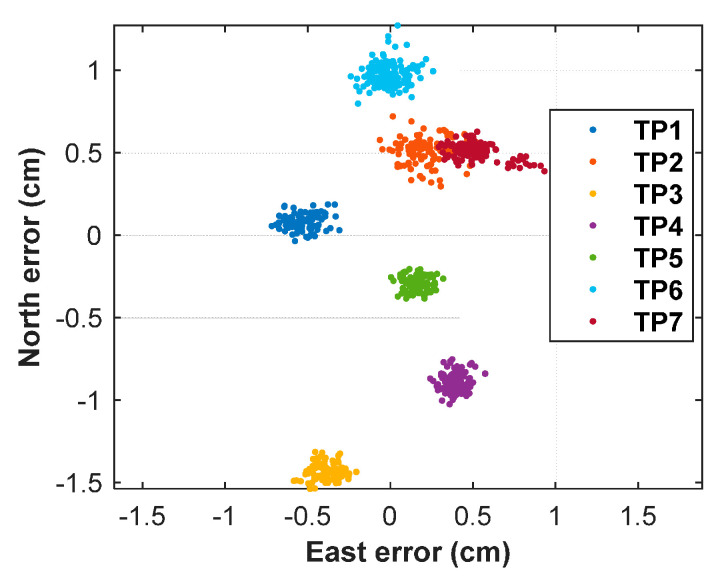
Positioning errors corresponding to seven TPs (test points).

The positioning errors corresponding to seven TPs are shown in Figure 15. The positioning accuracy statistics of the mean, root mean square (RMS), and standard deviation (STD) values are given in Table 8. The STDs are smaller than 0.15 cm, and the RMSs range from 0.343~1.487 cm for all TPs. The maximum of the positioning errors is 1.61 cm. The positioning biases are most likely caused by the multipath error and the antenna phase center offsets.

**Figure 16:**
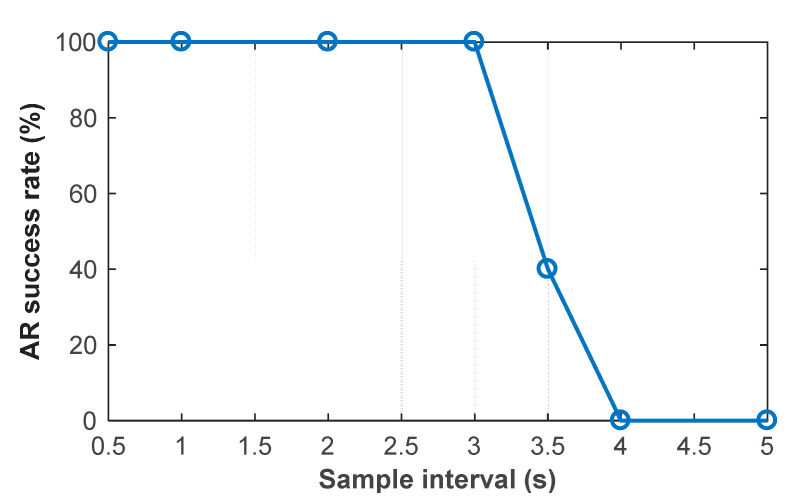
Statistical success rates as function of the sample interval.

The accuracy of predicted ‘float solution’ deduced from the Doppler-based velocity depends on the extrapolation time. The longer extrapolation time results in increasing the accumulated errors due to user dynamics. The extrapolation time is equal to the sample interval of the carrier phase measurement. Thus, using different sample intervals of the carrier phase measurement to investigate the influence of the Doppler-based velocity on the AR performance of the proposed kinematic ME-MAFA method. The statistical success rates as function of the sample interval are shown in Figure 16. With the sample interval increases, the success rate decreases clearly as expected. Because of the low dynamics of the toy train moving in an indoor environment, there is no loss of the success rate with 3 s sample interval. However, for a moving car in road testing, 4 Hz sample rate is needed when the Doppler-based velocity is used to aid AR [29].

## 6. Conclusions and Future Work

In precise single-point positioning of the pseudolite system, the carrier phase observations may suffer from cycle slips due to the dense multipath or the low-elevation pseudolite. A robust and instantaneous algorithm, the kinematic ME-MAFA, for OTF AR is proposed. With the proposed approach, carrier phase measurements and Doppler measurements are used for multi-epoch ambiguity determination. The kinematic ME-MAFA improves the model strength, compared with conventional single-epoch MAFA, increasing the success rate of ambiguity estimation effectively.

The efficiency and the performance of the proposed method are validated by numerical simulations and a semi-indoor experiment. The candidates are decreased by 25.15% and 37.2% on average in the simulations and in the real-world experiment respectively, making use of Doppler-based velocity information for predicting the ‘float position’ of the next epoch. Both simulated data and real data with artificial cycle slips are processed, and results indicate the proposed method is resistant to the cycle slips, similar to conventional AFM and MAFA.

During the round trip of the kinematic experiment, the integer ambiguities of all test groups are estimated correctly, and the actual success rate is 100%. The positioning accuracy is achieved better than 1.7 centimeters by our pseudilite system.

The simulation results show that the success rate of kinematic ME-MAFA is dominated by the measurement quality and geometry change. With the 5 Hz sampling rate of carrier phase measurement, 3.8 seconds on average of data are required for reliably ambiguities fixing in the experiment environment, with the threshold value of fail rate set to 5 × 10^-9^. The average computation time for searching the correctly fix solution is 26.1 seconds.

The computation time of using kinematic ME-MAFA to determine integer ambiguities is relatively long (dozens of seconds) because of the large-size search grids due to the poor accuracy of priori position. In the future work, tailored genetic algorithm, improved particle swarm optimization algorithm, or segmented simulated annealing algorithm can be put forward to reduce the computation load, referring to previous research for improving the single-epoch AFM or MAFA.

## Figures and Tables

**Table 1:** Success rates for the kinematic ME-MAFA, as function of the phase noise levels and the circle radius of the search region.

Noise Level [Cycle]	Search Radius [m]			
	1	2	4	6
0.03	99.73%	99.73%	99.82%	99.73%
0.04	97.75%	97.46%	97.79%	97.67%
0.05	92.92%	92.88%	92.60%	92.62%

**Table 2:** Comparison of the number of the Voronoi cells with the number of original grids in the search circle.

Search Radius [m]	**No. of Original Grids**	**Mean No. of Voronoi Cells**
1	341	257
2	1361	1029
4	5449	4067
6	12,281	9059

**Table 3:** Success rates with cycle slips.

Noise Level [Cycle]	Without Cycle Slip		With Cycle Slip	
	Kinematic ME-MAFA	LAMBDA	Kinematic ME-MAFA	LAMBDA
0.03	99.769%	99.733%	99.769%	0%
0.04	97.871%	97.569%	97.871%	0%
0.05	93.019%	92.510%	93.019%	0%

**Table 4:** Success rates as function of the geometry change.

Moving Distance [m]	Number of Epochs	Theoretical Lower Bound [%]	Statistical Value [%]	Theoretical Upper Bound [%]
1	6	55.312%	57.987%	100.000%
1.5	9	86.882%	88.646%	100.000%
2	11	97.419%	97.792%	100.000%
3	16	99.991%	99.993%	100.000%
4	21	100.000%	100.000%	100.000%

**Table 5:** Statistical parameter of fail rates as function of the geometry change.

Moving Distance [m]	Theoretical Fail Rates	Statistical Fail Rates	
		Mean	STD
1	4.47 × 10^−1^	3.66 × 10^−1^	9.70 × 10^−2^
2	2.58 × 10^−2^	2.56 × 10^−2^	1.82 × 10^−3^
3	9.19 × 10^−5^	9.19 × 10^−6^	2.39 × 10^−5^
4	4.92 × 10^−9^	4.92 × 10^−9^	1.79 × 10^−10^

**Table 6:** Coordinates of six pseudolites.

PL ID	East (m)	North (m)	Up (m)
PL1	−37.312	7.458	6.229
PL2	−27.191	−10.056	5.861
PL3	−28.520	8.107	6.155
PL4	−44.678	11.316	6.059
PL5	−46.102	6.879	7.224
PL6	−35.961	−10.678	6.175

**Table 7:** AR performance with artificial cycle slips.

Epoch	Pseudolite	Cycle Slip	Correct AR (Yes/No)
8	PL1	1	Yes
46	PL3	−2	Yes
91	PL6	3	Yes
125	PL2	−1	Yes
167	PL5	2	Yes
204	PL4	−3	Yes

**Table 8:** Statistics of position results of seven TPs.

	TP1	TP2	TP3	TP4	TP5	TP6	TP7
MEAN (cm)	0.532	0.549	1.485	0.973	0.334	0.974	0.717
STD (cm)	0.099	0.147	0.091	0.081	0.0761	0.117	0.136
RMS (cm)	0. 541	0.568	1.487	0.976	0.343	0.981	0.729
